# Bild Conception of Scientific Theory Structuring in Classical and Quantum Physics: From Hertz and Boltzmann to Schrödinger and De Broglie

**DOI:** 10.3390/e25111565

**Published:** 2023-11-20

**Authors:** Andrei Khrennikov

**Affiliations:** International Center for Mathematical Modeling in Physics and Cognitive Sciences, Linnaeus University, SE-351 95 Växjö, Sweden; andrei.khrennikov@lnu.se

**Keywords:** Bild conception, scientific theory, Helmholtz, Hertz, Boltzmann, Schrödinger, De Broglie, quantum mechanics, Brownian motion, Bell, prequantum classical statistical field theory

## Abstract

We start with a methodological analysis of the notion of scientific theory and its interrelation with reality. This analysis is based on the works of Helmholtz, Hertz, Boltzmann, and Schrödinger (and reviews of D’Agostino). Following Helmholtz, Hertz established the “Bild conception” for scientific theories. Here, “Bild” (“picture”) carries the meaning “model” (mathematical). The main aim of natural sciences is construction of the causal theoretical models (CTMs) of natural phenomena. Hertz claimed that a CTM cannot be designed solely on the basis of observational data; it typically contains hidden quantities. Experimental data can be described by an observational model (OM), often based on the price of acausality. CTM-OM interrelation can be tricky. Schrödinger used the Bild concept to create a CTM for quantum mechanics (QM), and QM was treated as OM. We follow him and suggest a special CTM for QM, so-called prequantum classical statistical field theory (PCSFT). QM can be considered as a PCSFT image, but not as straightforward as in Bell’s model with hidden variables. The common interpretation of the violation of the Bell inequality is criticized from the perspective of the two-level structuring of scientific theories. Such critical analysis of von Neumann and Bell no-go theorems for hidden variables was performed already by De Broglie (and Lochak) in the 1970s. The Bild approach is applied to the two-level CTM-OM modeling of Brownian motion: the overdamped regime corresponds to OM. In classical mechanics, CTM=OM; on the one hand, this is very convenient; on the other hand, this exceptional coincidence blurred the general CTM-OM structuring of scientific theories. We briefly discuss ontic–epistemic structuring of scientific theories (Primas–Atmanspacher) and its relation to the Bild concept. Interestingly, Atmanspacher as well as Hertz claim that even classical physical theories should be presented on the basic of two-level structuring.

## 1. Introduction

The Bild conception of scientific theory was developed by Hertz [1,2], starting with Helmholtz analysis [3,4,5,6] of interrelation between physical reality and scientific theory. This line of thinking was continued by Boltzmann [7,8] and in the 1950s by Schrödinger [9,10,11,12,13,14,15]. The articles of D’Agostino [16,17,18,19,20,21] contain philosophically deep reviews on their works.

Typically, the German word “Bild” is translated to English as “picture”. However, for the Bild conception in science, the meaning “model conception” is more appropriate; see also Patton [22].

Bild is translated variously in the literature as “picture”, “image”, or “model”. A Bild should not be understood as a visual or mental image or picture, however. I tend to use the German word to underscore the fact that Hertz and Wittgenstein use the same terminology.

Patton also pointed out that the Bild conception is a precursor of model theory that influenced Einstein, Hilbert, and Schrödinger.

Helmholtz pointed out that a scientific theory does not describe reality as it is. A scientific theory structures our sensations and perceptions within a priori forms of intuition (cf. with Kant). Such structuring leads to models of reality reflecting some features of the environment of observers. Therefore, the dream for creation of a “true theory” matching perfectly with natural phenomena is in contradiction with Helmholtz’s philosophy of science. Hertz, Boltzmann, and Schrödinger were the followers of Helmholtz, and for them the Bild conception was not about the pictures of reality but about creation of models of reality. The term “picture” preassumes that this is a picture of something existing in reality. However, due to the Bild conception, the human mind constructs a model of natural phenomena. In any event, Helmholtz’s philosophic position supports the use of the English word “model” in the presentation of the Bild conception.

Observational data should be considered with caution. Helmholtz highlighted *causality* of the natural phenomena, and for him the main task of a scientific theory is to reflect this causality. Thus, from his viewpoint, the main aim of scientific studies is construction of the *causal theoretical models* (CTMs) of natural phenomena. Theoretical causality is an image of natural causality. In terms of cognition, causality of human reasoning reflects causality of natural processes, and it was developed during biological evolution, from the primitive forms of life to humans.

We remark that causality is a complex notion with deep philosophic and physical counterparts. The viewpoint presently used in physics on causality was formed under the strong influence of the development of special relativity. Helmholtz’s works were written earlier and he, as well as Hertz and Boltzmann, used a more general viewpoint on causality (see Section 2 for the discussion).

Hertz followed Helmholtz’s approach to scientific theory, but he claimed that, generally, CTM cannot be designed solely on the basis of observational data and it typically contains hidden quantities. So, in physics, hidden variables were employed long before the quantum revolution. Experimental data are described by an observational model (OM), which is often acausal. The CTM-OM interrelation can be tricky. Hertz presented this framework [1,2] as a Bild conception (model conception). He highlighted the role of mathematics and treatment of a scientific model as a mathematical model (see also Plotnitsky [23]). In particular, Hertz presented Maxwell’s theory as the system of the Maxwell equations.

Later, the Bild conception was resurrected in the foundational studies of Schrödinger [9,10,11,12,13,14,15] (see especially [9]), who tried to create CTM for quantum mechanics (QM), and QM was treated as OM. He advertised the two-level structuring of the description of microphenomena. We follow him and suggest a special CTM for QM, so-called *prequantum classical statistical field theory* (PCSFT) [24,25,26]. QM treated as OM can be considered as a PCSFT image, but not as straightforward as in Bell’s model with hidden variables [27,28].

We analyze Bell’s model with hidden variables within the Bild framework and criticize identification of subquantum (hidden) quantities with quantum observables and hidden probability distributions with quantum probability distributions. The evident barrier for such identification is the Heisenberg uncertainty principle (and the Bohr complementarity principle [23,29,30,31,32,33,34,35]). The same viewpoint was presented long ago by De Broglie [36] (see also Lochak [37,38,39]), who justified the legitimacy of his double solution theory [40,41], in fact within the Bild conception (although it seems that he was not aware of it). He pointed to the inconsistency of the no-go interpretation of the von Neumann [42] and Bell [27,28] theorems. The De Broglie double solution model is a CTM for QM. Its structuring within the Bild conception deserves a separate article as well as the Bild conception presentation of Bohmian mechanics.

This is a good place to note that one should not identify De Broglie’s and Bohm’s theories, nor consider the latter as just an extension and improvement of the former. De Broglie did not consider his theory as nonlocal; he noted that its nonlocality is only apparent; this is nonlocality of mathematical equations and not physical processes [40]. Deep foundational studies on De Broglie’s double solution model are presented in the works of Bacciagaluppi, e.g., [43,44]; see also the Appendix A written by Bacciagaluppi and Valentini in [41].

We also use the Bild approach for the two-level CTM-OM modeling of Brownian motion: the overdamped regime corresponds to OM [45]. Coarse-grained velocities are observable quantities. This example represents clearly the physical origin of the two-level structuring of the mathematical description of Brownian motion. This is the timescale separation technique. The evolution of the momenta of the Brownian particles is very fast and cannot be resolved on the timescales available to the experiment. We notice that the OM model for the Brownian motion shows some distinguished properties of QM; see, e.g., article [45] for the corresponding uncertainty relations and Brownian entanglement theory.

The idea of timescale separations is one of the most pertinent ones in nonequilibrium statistical physics. In a qualitative form, it appears already in good textbooks on this subject [46,47] and has since then been formalized in various contexts and on various levels of generality [48,49,50,51,52,53].

In classical mechanics, CTM=OM; on the one hand, this is very convenient; on the other hand, this exceptional coincidence blurred the general CTM-OM structuring of scientific theories.

We also briefly discuss ontic–epistemic structuring of scientific theories (Primas–Atmanspacher [54,55]; see also artciles [56,57]) and its relation to the Bild concept.

This paper is a continuation of my works [25,26]. I hope that in this paper the Bild conception and its implementation for quantum and classical mechanics are presented clearly. Presentation of the two-level CTM-OM description for Brownian motion is a good complement to such description for quantum phenomena. The CTM-OM viewpoint on the Bell inequality project clarifies the difference in the positions of Schrödinger [9,10,11,12,13,14,15] and Bell [27,28] on the possibility to construct a subquantum model with hidden variables.

## 2. From the Bild Conception to Two-Level Structuring of Scientific Theories

### 2.1. Von Helmholtz: Scientific Theory Is Not a Faithful Image of Nature

We start by citing the article of D’Agostino [21]: Hermann von Helmholtz (1821–1894) was one of the first scientists to criticize the objective conception of physical theory by denying that theoretical concepts describe real physical objects. He realized that Immanuel Kant’s a priori forms of intuition should to be taken into account in analyzing problems that were emerging at the end of the nineteenth century in the new formulations of physics.

The objective conception of physical theory also was criticized by such physicists as Heinrich Hertz (1857–1894) and Ludwig Boltzmann (1844–1906), who adopted the Kantian term Bild to designate the new conception of physical theory, which they took to mean not a faithful image of nature but an intellectual construct whose relationship to empirical phenomena was to be analyzed.

The works of von Helmholtz, Hertz, and Boltzmann [1,2,3,7,8] played a crucial role in the development of a novel scientific methodology. Since the times of Galileo and Newton, scientific theories have varied essentially in their content, but nobody has questioned their “ontological significance”, their adequacy to represent physical reality.

In 1878, von Helmholtz posed the following philosophical questions [3]:

What is true in our intuition and thought? In what sense do our representations correspond to actuality?

Von Helmholtz’s answers to these questions were based on his physiological, especially visual, research that led him to the following conclusion [3]:

Inasmuch as the quality of our sensation gives us a report of what is peculiar to the external influence by which it is excited, it may count as a symbol of it, but not as an image. For from an image one requires some kind of alikeness with the object of which it is an image …. We point out that, if the fathers of QM would take this statement into account, then their surprise regarding “unusual features” of the quantum mechanical description of microphenomena would not be so strong. We also note that Bohr’s views on QM match with this conclusion of Helmholtz. Surprisingly, it seems that Bohr had never referred to his works.

Helmholtz’s viewpoints on interrelation of sensations and general observations and real objects led to the well-known statement on the parallelism of the laws of nature and science [3]:

Every law of nature asserts that upon preconditions alike in a certain respect, there always follow consequences which are alike in a certain other respect. Since like things are indicated in our world of sensations by like signs, an equally regular sequence will also correspond in the domain of our sensations to the sequence of like effects by the law of nature [that like effects follow from] … like causes.

### 2.2. Causality

We point out that Helmholtz’s statement that, upon alike preconditions in a certain respect, there always follow consequences that are alike in a certain other respect is about physical causality. So, for Helmholtz, nature is causal; i.e., laws in nature really exist and laws presented in scientific theories are mental representations of laws of nature. The laws expressed by our sensation and through them by our perception are “parallel” to natural laws, but only parallel, not identical, since our mind operates not with precise images of real objects but only with symbols assigned to them.

In modern physics, the notion of causality was strongly influenced by the creation of relativity theory. An effect can occur only from a cause that is in its past light cone, and a cause can lead only to an effect inside its future light cone. This viewpoint on causality was not present in times of Helmholtz and Hertz.

In philosophy, the notion of causality is based on the categories of the cause and effect formalizing the genetic connection between events (or states) such that one event (or state) induces another event (or state)—an effect (e.g., [58]). A few causes can generate the same effect.

Many philosophers claim that causality is a primary notion preceding the notions of space and time; see, e.g., Robb [59] and Whitehead [60].

In the present paper, we consider the special form of causality that is mathematically described via the functional representation,

(1) y=f(x),

where *x* and *y* are interpreted as the cause and effect variables (see [61]). The cause–effect interpretation is crucial in mathematical physics, not arbitrary; Equation (1) contains the causal meaning.

In coming considerations, we select the *x*-variable as the system’s state. The latter is interpreted as a context, a complex of physical conditions (see [62]). A physical quantity *A* is mathematically represented by a function $f_{A}.$ A state plays the role of preconditions in the above citation of Helmholtz. Then, these preconditions, context, contribute to generation of the *A* value via (1); this value generation is an event. A state precedes this event; function $f_{A}$ is considered as effect generator. For example, *x* is sensation and $y=f_{p}\left(x\right)$ the corresponding perception.

Consider the classical phase space model. Here, states are provided by points of the phase space, x=(q,p). Consider the Hamilton function $H\left(q,p\right)=p^{2}/2m+V\left(q\right).$ Then, x=(q,p) is the cause and y=H(q,p) is the effect—the event of assigning energy to a system in this state. In this framework, it is meaningless to speak about the energy quantity without specifying the system’s state, the context for energy determination. The Hamilton function describes generation of the energy-value events from states.

### 2.3. Hertz: Need of Hidden Quantities

Hertz questioned Helmholtz’s parallelism of laws. Hertz believed that Helmholtz’s parallelism of laws was not only indeterminate but in general even impossible if theory were limited to describing observable quantities [2]:

If we try to understand the motions of bodies around us, and to refer them to simple and clear rules, paying attention only to what can be directly observed, our attempt will in general fail. We soon become aware that the totality of things visible and tangible do not form an universe conformable to law, in which the same results always follow from the same conditions. We become convinced that the manifold of the actual universe must be greater than the manifold of the universe which is directly revealed to us by our senses.

For Hertz, a causal theory cannot be based solely on observable quantities [2]: they do not form a universe conformable to law in which the same results always follow from the same conditions. Only by introducing hidden quantities can Helmholtz’s parallelism of laws become a general principle in physical theory. However, such hidden quantities (concepts that correspond to no perceptions) precipitate too much freedom regarding choice of theoretical concepts. To limit this freedom of choice, Hertz introduced special requirements for the validation of a physical theory. Aside from causality, the most important was the theory’s simplicity [2]:

It is true we cannot a priori demand from nature simplicity, nor can we judge what in her opinion is simple. But with regard to images [Bilder] of our own creation we can lay down requirements.We are justified in deciding that if our images are well adapted to the things, the actual relations of the things must be represented by simple relations between the images.

So, Helmholtz and Hertz questioned the ontological status of scientific theories as describing reality as it is. Scientific theories are only “Bilder” models of reality. Outputs of sensations and observations are just symbols encoding external phenomena. Hence, one should not sanctify observational quantities and their role in scientific theories. Moreover, an observational theory, i.e., operating with solely observables, cannot be causal. Causality demands introduction of hidden (unobservable) quantities. Of course, a theory with hidden quantities should be coupled to observational data. However, this coupling need not be straightforward.

According to Helmholtz, a scientific theory should be causal. Hertz claimed [2] that, generally, the causality constraint requires invention of hidden quantities; a causal description cannot be completed solely in terms of observational quantities. This approach unties scientists’ hands by introducing hidden quantities so that they can generate a variety of theoretical causal models coupled to the same observational quantities. How can one select a “good” causal model? Hertz suggested to use the model’s simplicity as a criterion for such selection. We note that even a “good model” does not describe reality as it is; it provides just a mathematical symbolic representation involving a variety of elements having no direct relation with the observational quantities.

### 2.4. Is It Possible to Construct the “True Model” Describing Reality as It Is?

It is natural to search for such a (causal) theoretical model that would describe what nature really is, a “true model” (an ontic model). It is not clear whether Hertz might hope to design such a model for the electromagnetic phenomenon. He tried to model it with systems of mechanical oscillators, i.e., to go beyond the electromagnetic field representation [1]. However, he did not succeed with this project. His project was not meaningless. It has some degree of similarity with the representation of the quantum electromagnetic field as a system of quantum oscillators—photons. Schrödinger, who later contributed to the development of the Bild concept of scientific theories, especially in the relation to the quantum foundations, claimed [10] that no true model can be formulated on the basis of our large-scale experience because

We find nature behaving so entirely differently from what we observe in visible and palpable bodies of our surroundings …. A completely satisfactory model of this type is not only practically inaccessible, but not even thinkable. Or, to be precise, we can, of course, think it, but however we think it, it is wrong; not perhaps quite as meaningless as a “triangular circle”, but much more so than a “winged lion”.

Creation of a causal theoretical model coupled to some observed natural phenomena is a complex and long process. Moreover, there is always a chance that such a model would never be found due to the intellectual incapacity of humankind. Therefore, it is natural to design models matching observations but not satisfying the causality constraint. We call such models *observational models.*

### 2.5. Observational vs. Causal Theoretical Model

Thus, we distinguish two classes of models, *observational models* (OMs) and *causal theoretical models* (CTMs). We remark that both kinds of scientific models are mental constructions, providing symbolic mathematical descriptions of natural phenomena. One may say that any model is theoretical, so OM is also theoretical, and he would be right. So, the main difference between OM and CTM is in causality. If OM is causal by itself, then there is no need to go beyond it with some CTM.

Interrelation between CTM and OM, $M_{C}$ and $M_{O},$ depends on the present stage of development of science. If $M_{C}$ rightly reflects the real physical processes, then development of measurement technology can lead to novel observational possibilities and some hidden quantities of $M_{C}$ can become measurable. Hence, $M_{C}$ becomes OM, $M_{C}\to M_{O}^{\prime }.$ In principle, $M_{O}^{\prime }$ need not cover all observations described by the previous OM $M_{O}.$ New theoretical efforts might be needed to merge $M_{O}$ and $M_{O}^{\prime }.$ This abstract discussion will be illustrated by the concrete example from classical statistical physics—the two-level modeling of Brownian motion (Section 10.1).

### 2.6. Schrödinger: Bild Conception Viewpoint on Quantum Mechanics

The ideas of Helmholtz and Hertz were further developed (and modified) in the works of Boltzmann [7,8]. Then, 60 years later, Schrödinger [9,10,11,12,13,14,15] contributed to the development of the Bild viewpoint on quantum theories. He confronted the special case of the aforementioned problem.

OM for microphenomena was developed (particularly due to his own efforts): this is QM. However, QM suffered from acausality. The impossibility to solve the measurement problem (which was highlighted by von Neumann [42]) generates a gap in the quantum description of microphenomena. Schrödinger came back to this problem in the 1950s [9,10,11,12,13,14,15]; this comeback was stimulated by the development of quantum field theory and the method of second quantization.

He saw in quantum field theory a possibility to justify his attempts regarding the purely wave (continuous) approach to modeling of microphenomena. In complete agreement with the Bild concept, he considered QM as an observational model. As well as von Neumann, Schrödinger highlighted its acausality. However, it was not treated as a property of nature as it is; i.e., quantum acausality (of measurements and spontaneous quantum events) is not ontic. We notice that, for von Neumann, it is ontic; he wrote about “irreducible quantum randomness” [42]. Quantum acausality is just a property of special OM—QM. Schrödinger claimed that quantum acausality is related to ignoring the Bild concept and assigning the ontological status to quantum particles; see his article “What is an elementary particle?” [9]. We remark that Bohr did not question the ontological status of quantum systems, atoms, electrons, and perhaps even photons [23,29,30,31,32,33,34,35]. Schrödinger considered the indistinguishability of quantum particles as a sign that they do not have an ontological status. Hence, instead of OM (=QM), one can hope to develop CTM for microphenomena by liberating it from particles and operating solely with waves.

Since waves propagate in space, for Schrödinger, causality (in fact, wave causality) is coupled to continuity in space, so the waves should be continuous (see Plotnitsky [23] on analysis of continuity vs. discontinuity in physics). We remark that he considered continuity of waves in multi-dimensional space $R^{3n}.$ In the 1920s, the fact that the multi-particle Schrödinger equation described the waves not in “the physical space” $R^{3},$ but in “the mathematical space” $R^{3n},$ was disturbing for him. This was the main reason for Schrödinger to accept the probabilistic interpretation of the wave function. At that time, he did not use the Bild concept for scientific theories (he was not aware about the works of Helmholtz, Hertz, and Boltzmann?). By the Bild concept, the wave representation of QM is just a symbolic mathematical representation of the microphenomena. The use of multi-dimensional space $R^{3n}$ has the same descriptive status as the use of $R^{3}.$

Schrödinger dreamed of the creation of CTM for microphenomena; his concrete intention was towards a wave-type model. He also highlighted the principle of continuity for “quantum waves”, but he suspected that it would be valid only at the micro level. He pointed to quantum field theory as a good candidate to proceed in this direction. Since he coupled causality and continuity, it became possible to relax the causality-continuity constraint and restrict this constraint to the level of infinitesimals. In a theoretical model completing QM (an observational model) for which Schrödinger dreamed, causality need not be global.

Schrödinger’s continuous wave completion project for QM has some degree of similarity with Einstein’s project on designing a classical field model of microphenomena, which he announced with Infeld in a popular form in a book [63]. Einstein’s intention was that a complete theory beyond QM should be nonlinear field theory. Later, Infeld contributed a great deal to this project. In contrast to Einstein, Schrödinger dreamed of a linear model.

However, Einstein did not appeal to the Bild concept on the two-level modeling of natural phenomena, observational and causal theoretical (OM and CTM), and a possible gap between these two models. The presence of such a gap, in particular, implies that CTM need not describe the observational data straightforwardly.

Einstein’s project on reconsideration of quantum foundations starting with the EPR paper [64] was not directed to the two-level structuring of the mathematical description of microphenomena. He dreamed of the creation of a CTM that would match perfectly with quantum observations. Einstein argued that the current theory of QM was incomplete. According to this interpretation, it is believed that quantum theory is incomplete as some variables are present in the theory that have not been known. Such variables are known as hidden variables. A special theory with hidden variables corresponding to Einstein’s dream was suggested by Bell [27,28]. Its main distinguishing property is identification of the outcomes of quantum observables with functions of hidden variables and, hence, identification of quantum and subquantum averages and correlations. This identification contradicts the Bild conception and the two-level structuring of physical theories. By the latter, quantities in a subquantum model with hidden variables are not straightforwardly coupled to quantum observables. In particular, such a viewpoint was advertised by De Broglie [36] (Section 9).

Schrödinger understood [10] that a CTM of microphenomena of the wave type is not the observed or observable facts; and still less do we claim that we thus describe what nature (matter, radiation, etc.) really is. In fact we use this picture (the so-called wave picture) in full knowledge that it is neither. This statement expresses the extreme view on the Bild concept; Schrödinger [10] also pointed out that observed facts…appear to be repugnant to the classical ideal of a continuous description in space and time. Such highlighting of decoupling of theory and observations was too provocative and played a negative role. The idea of using the Bild concept in quantum foundations was rejected by the majority of experts in quantum foundations.

### 2.7. Primas and Atmanspacher: Ontic–Epistemic Modelling

However, the Bild concept did not disappear completely and its traces can be found in the philosophy of the ontic–epistemic structuring of physical theories that was developed by Primas and Atmanspacher [54] (see also, e.g., [56,57]). They tried to find an answer [56] to the old question:

Can nature be observed and described as it is in itself independent of those who observe and describe—that is to say, nature as it is “when nobody looks”?

As well as Helmholtz, Hertz, Boltzmann, and Schrödinger, they pointed out that observations provide to observers only some knowledge about systems; this knowledge is incomplete. This knowledge is mathematically structured within an epistemic (=observational) model. For them, QM is such a model; i.e., w.r.t. QM, the views of Schrödinger and Primas–Atmanspacher coincide. Then, in the same way as Schrödinger, they want to obtain a complete model of microphenomena. The crucial difference from the Bild concept is that Primas and Atmanspacher were seeking an ontic model, a model of reality as it is, the “true model” in terms of Schrödinger. Generally, Primas and Atmanspacher also supported the idea of the two-level structure of scientific theories: epistemic (observational) and ontic. As well as Schrödinger, they pointed out that the connection between epistemic and ontic models is not straightforward. Causality is the basic property of the ontic model. So, if one would ignore the term “ontic”, then, formally (and mathematically), Primas–Atmanspacher structuring of the scientific description of nature is similar to the Bild concept. In contrast to Schrödinger, they did not emphasize the continuous wave structure of an ontic model beyond QM.

However, by pointing to formal mathematical similarity of the ontic–epistemic and Bild approaches, one should remember that they differ crucially from the foundational perspective. We recall [56] that

Ontological questions refer to the structure and behavior of a system as such, whereas epistemological questions refer to the knowledge of information gathering and using systems, such as human beings.

From the Bild perspective, it is totally meaningless even to refer to the structure and behavior of a system as such…The essence of the ontic–epistemic approach is expressed in the following quote from Atmanspacher [56] (for more details, the reader is referred to Primas [55]):

Ontic states describe all properties of a physical system exhaustively. (“Exhaustive” in this context means that an ontic state is “precisely the way it is”, without any reference to epistemic knowledge or ignorance.) Ontic states are the referents of individual descriptions, the properties of the system are treated as intrinsic properties. Their temporal evolution (dynamics) is reversible and follows universal, deterministic laws. As a rule, ontic states in this sense are empirically inaccessible. Epistemic states describe our (usually non-exhaustive) knowledge of the properties of a physical system, i.e., based on a finite partition of the relevant phase space. The referents of statistical descriptions are epistemic states, the properties of the system are treated as contextual properties. Their temporal evolution (dynamics) typically follows phenomenological, irreversible laws. Epistemic states are, at least in principle, empirically accessible.

From the Bild perspective, the statement that ontic states are the referents of individual descriptions; the properties of the system are treated as intrinsic properties is meaningless since systems do not have intrinsic properties; a theoretical causal model beyond the quantum observational (epistemic) model still describes not the properties of the systems but our mental pictures.

In short, for Primas and Atmanspacher, a CTM describes reality as it is; for them, by searching a CTM, one searches for a true model of reality. From the Bild viewpoint, no CTM describes reality as it is; therefore, Schrödinger was against searching for a “true CTM” beyond QM.

### 2.8. Nietzsche

Moreover, we conclude this section by the quote from Nietzsche (written in 1873, but published later); his statement is very similar to Helmholtz’s statements, but it is more passionate or even poetic! It seems that Nietzsche was influenced by Helmholtz, especially on nerve stimulus. Nietzsche wrote about language, but the point is more general [65]:

The various languages placed side by side show that with words it is never a question of truth, never a question of adequate expression; otherwise, there would not be so many languages. The “thing in itself” (which is precisely what the pure truth, apart from any of its consequences, would be) is likewise something quite incomprehensible to the creator of language and something not in the least worth striving for. This creator only designates the relations of things to men, and for expressing these relations he lays hold of the boldest metaphors. To begin with, a nerve stimulus is transferred into an image: first metaphor. The image, in turn, is imitated in a sound: second metaphor. And each time there is a complete overleaping of one sphere, right into the middle of an entirely new and different one. One can imagine a man who is totally deaf and has never had a sensation of sound and music. Perhaps such a person will gaze with astonishment at Chladni’s sound figures; perhaps he will discover their causes in the vibrations of the string and will now swear that he must know what men mean by “sound”. It is this way with all of us concerning language; we believe that we know something about the things themselves when we speak of trees, colors, snow, and flowers; and yet we possess nothing but metaphors for things-metaphors which correspond in no way to the original entities. In the same way that the sound appears as a sand figure, so the mysterious of the thing in itself first appears as a nerve stimulus, then as an image, and finally as a sound. Thus the genesis of language does not proceed logically in any case, and all the material within and with which the man of truth, the scientist, and the philosopher later work and build, if not derived from never-never land, is a least not derived from the essence of things.

## 3. Coupling of Theoretical and Observational Models

Models explored in natural science are mainly mathematical. Therefore, coupling between CTM and OM corresponding to the same natural phenomena is a mapping of one mathematical structure to another.

Let M equal some mathematical model of natural phenomena, either CTM or OM. It is typically based on two spaces, the space of states *S* and the space of quantities V. For OM, *V* is the space of observables; instead of states, one can consider measurement contexts.

Consider OM model $M_{O}=\left(S_{O},V_{O}\right)$ and its causal theoretical completion $M_{C}=\left(S_{C},V_{C}\right).$ It is natural to have a mathematical rule establishing correspondence between them. We recall that CTMs are causal and OMs are often acausal; if it happens that OM is causal, then there is no need for a finer description given by some CTM. Thus, the task is to establish correspondence between causal and acausal models. It is clear that such correspondence cannot be straightforward. We cannot map directly states from $S_{C}$ to states from $S_{O}.$ Causality can be transformed into acausality through consideration of probability distributions. So, consider some space of probability distributions $P_{C}$ on the state space $S_{C}$ and construct a map from $P_{C}$ to $S_{O},$ the state space of OM. This approach immediately implies that the states of OM are interpreted statistically. We should also establish correspondence between quantities of $M_{C}$ and $M_{O}.$ Thus, we need to define two physically natural maps:

(2) $$J_{S}:P_{C}\to S_{O},\;J_{V}:V_{C}\to V_{O}.$$

Since $J_{S}$ is not defined for states of CTM but only for probability distributions, “physically natural” means coupling between the probability structures of $M_{C}$ and $M_{O};$ the minimal coupling is the equality of averages

(3) $${\langle J_{V}\left(f\right)\rangle }_{J_{S}\left(P\right)}={\langle f\rangle }_{P}$$

and correlations between quantities

(4) $${\langle J_{V}\left(f\right)J_{V}\left(g\right)\rangle }_{J_{S}\left(P\right)}={\langle fg\rangle }_{P}.$$

Generally, the correlation need not be defined, so (4) should hold for quantities $f,g\in V_{C}$ and observables $A_{f}=J_{V}\left(f\right)$ and $A_{g}=J_{V}\left(g\right)$ for which the correlations in the states *P* and $J_{S}\left(P\right)$ are defined.

As was pointed out in Section 2.2, in this paper, we consider the mathematical description of causality by functional Equation (1). Therefore, we assume that $V_{C}$ can be represented as a space of functions $f:S_{C}\to R.$ Such model is causal; the state ϕ uniquely determines the values of all quantities belonging $V_{C}:\phi \to f\left(\phi \right).$ The state space $S_{C}$ can be endowed with a σ-algebra of subsets F. Elements of $P_{C}$ are probability measures on F. The minimal mathematical restriction on elements of $V_{C}$ is that they are measurable functions, $f:S_{C}\to R.$ In such a framework,

(5) $${\langle f\rangle }_{P}=\int_{S_{C}}f\left(\lambda \right)P\left(d\lambda \right),{\langle fg\rangle }_{P}=\int_{S_{C}}f\left(\lambda \right)g\left(\lambda \right)P\left(d\lambda \right),$$

if the integrals exist, e.g., if CTM quantities are square integrable:

$$\int_{S_{C}}{\left|f\left(\lambda \right)\right|}^{2}P\left(d\lambda \right)<\infty .$$

Since in $M_{O}$ quantities have the experimental statistical verification, we establish some degree of experimental verification for $M_{C}$ through mapping of $M_{C}$ to $M_{O}.$ However, such verification is only indirect; one should not expect direct coupling between quantities of $M_{C}$ and experiment (as Einstein, Bell, and all their followers wanted to obtain). Generally, these maps are neither one-to-one nor onto.

- A cluster of probability distributions on $S_{C}$ can be mapped into the same state from $S_{O}.$
- $J_{S}\left(P_{C}\right)$ need not coincide with $S_{O}.$
- A cluster of elements of $V_{C}$ can be mapped into a single variable (observable) from $V_{O}.$
- $J_{V}\left(V_{C}\right)$ need not coincide with $V_{O}.$

Moreover, the model-correspondence maps $J_{S},J_{V}$ need not be defined on whole spaces $P_{C}$ and $V_{C}.$ They have their domains of definition, $D_{J_{S}}\subset P_{C}$ and $D_{J_{V}}\subset V_{C}.$ (In principle, one can reduce $P_{C}$ to $P_{C}^{\prime }=D_{J_{S}}$ and $V_{C}$ to $V_{C}^{\prime }=D_{J_{V}}$ and operate with maps $J_{S},J_{V}$, which are defined everywhere on these reduced spaces of CTM’s states and quantities.)

We remark that the same $M_{O}$ can be coupled to a variety of CTMs. We also remark that the same observational data can be mathematically described by a variety of OMs.

We also remark that, similar to the deformation quantization (here, we discuss just the mathematical similarity), CTM may depend on some small parameter κ (in the deformation quantization, this is action, roughly speaking the Planck constant h). Thus, $M_{C}=M_{C}\left(\kappa \right).$ In such more general framework, the correspondence maps also depend on κ; i.e.,  $J_{S}=J_{S}\left(\kappa \right),J_{V}=J_{V}\left(\kappa \right).$ The probabilistic coupling constraints (3), (4) can be weakened:

(6) $${\langle J_{V}\left(\kappa ;f\right)\rangle }_{J_{S}\left(\kappa ;P\right)}={\langle f\rangle }_{P}+o\left(\kappa \right),\kappa \to 0,$$

(7) $${\langle J_{V}\left(\kappa ;f\right)J_{V}\left(\kappa ;g\right)\rangle }_{J_{S}\left(\kappa ;P\right)}={\langle fg\rangle }_{P}+o\left(\kappa \right),\kappa \to 0$$

(see [24,25,66,67,68]). The problem of identification of the parameter κ with some physical scale is complex (see, e.g., [66,67] for an attempt of such identification within PCSFT).

## 4. Prequantum Classical Statistical Field Theory as a Causal Theoretical Model for Quantum Mechanics

We illustrate the general scheme of CTM-OM correspondence by two theories of microphenomena, QM as $M_{O}$ and PCSFT as $M_{C}.$ Re-denote these model with the symbols $M_{\mathrm{QM}}$ and $M_{\mathrm{PCSFT}}.$ We briefly recall the basic elements of PCSFT (see [24,25,26] for details).

In $M_{QM}$, states are given by density operators acting in complex Hilbert space H (with scalar product 〈·|·〉) and observables are represented by Hermitian operators in H. Denote the space of density operators by $S_{\mathrm{QM}}$ and the space of Hermitian operators by $V_{\mathrm{QM}}.$

In $M_{\mathrm{PCSFT}}$, states are vectors of H; i.e.,  $S_{\mathrm{PCSFT}}=H.$ Physical quantities are quadratic forms

$$\phi \to f\left(\phi \right)=\langle \phi |\hat{A}|\phi \rangle ,$$

where $\hat{A}\equiv {\hat{A}}_{f}$ is a Hermitian operator. The space of quadratic forms is denoted by the symbol $V_{\mathrm{PCSFT}}.$ Consider probability measures on the σ-algebra of Borel subsets of H (i.e., generated by balls in this space) having zero first momentum; i.e.,

(8) $$\int_{H}\langle \phi |a\rangle dp\left(\phi \right)=0$$

for any vestor a∈H, and finite second momentum; i.e.,

(9) $$E_{p}\equiv \int_{H}{∥\phi ∥}^{2}dp\left(\phi \right)<\infty .$$

Denote the space of such probability measures by the symbol $P_{\mathrm{PCSFT}}.$

Random vectors are defined on some Kolmogorov probability space (Ω,F,P); these are functions ϕ:Ω→H, which are measurable w.r.t. to the Borel σ-algebra of H; i.e., for any Borel subset *B* of H,$\phi^{-1}\left(B\right)\in F.$ A map is measurable iff, for any c>0, the set $\Omega_{\phi ,c}=\left\{\omega \in \Omega :||\phi \left(\omega \right)||<c\right\}\in F.$

Moreover, we can start not with probability measures but with H-valued random vectors with zero mean value and finite second moment: ϕ=ϕ(ω), such that E[ϕ]=0 and $E[∥\phi {∥}^{2}]<\infty .$ The space of such random vectors is denoted by the symbol $R_{\mathrm{PCSFT}}.$ In the finite-dimensional case, these are complex vector-valued random variables; if H is infinite-dimensional, then the elements of $R_{\mathrm{PCSFT}}$ are random fields.

An example of random fields is given by selection $H=L_{2}\left(R^{n};C\right)$ of square integrable complex valued functions. Each $M_{C}$ state ϕ is an $L_{2}$-function, $\phi :R^{n}\mapsto C.$ Random fields belonging to $R_{\mathrm{PCSFT}}$ are functions of two variables, ϕ=ϕ(x;ω): chance parameter ω and space coordinates x.

We remark that, for the state space $H=L_{2}\left(R^{n};C\right),$ the quantity $E_{p}$ can be represented as

$$E_{p}=\int_{H}E\left(\phi \right)dp\left(\phi \right),$$

where

$$E\left(\phi \right)={∥\phi ∥}^{2}=\int_{R^{n}}{\left|\phi \left(x\right)\right|}^{2}dx$$

is the energy of the field. The quantity $E_{p}$ can be interpreted as the average of the field energy with respect to the probability distribution *p* on the space of fields. We can also use the random field representation. Let ϕ=ϕ(x;ω) be a random field. Then, its energy is the random variable

$$E_{\phi }\left(\omega \right)=\int_{R^{n}}{\left|\phi \left(x;\omega \right)\right|}^{2}dx$$

and $E_{p}$ is its average.

For any $p\in P_{\mathrm{PCSFT}},$ its (complex) covariance operator ${\hat{B}}_{p}$ is defined by its bilinear (Hermitian) form:

(10) $$\langle a|{\hat{B}}_{p}|b\rangle =\int_{H}\;\langle a|\phi \rangle \langle \phi |b\rangle \;dp\left(\phi \right),\;a,b\in H,$$

or, for a random field ϕ, we have

$$\langle a|{\hat{B}}_{\phi }|b\rangle =E\left[\langle a|\phi \rangle \langle \phi |b\rangle \right].$$

We note that

(11) $$E_{p}=\int_{H}{∥\phi ∥}^{2}dp\left(\phi \right)=\mathrm{Tr}{\hat{B}}_{p}$$

or in terms of a random field:

(12) $$E_{p}={E[||\phi ||}^{2}]=E[\int_{R^{n}}{\left|\phi \left(x;\omega \right)\right|}^{2}dx]=\mathrm{Tr}{\hat{B}}_{p}.$$

Thus, the average energy of a random field ϕ=ϕ(ω,x) can be expressed via its covariance operator.

Generally, a probability measure (H-valued random variable) is not determined by its covariance operator (even under the constraint given by zero average).

A complex covariance operator has the same properties as a density operator aside from normalization by the trace one; a covariance operator ${\hat{B}}_{p}$ is

- Hermitian;
- positively semidefinite;
- trace class.

A “physically natural coupling” of the models $M_{\mathrm{QM}}$ and $M_{\mathrm{PCSFT}}$ is based on the following formula mathematically coupling the averages for these models. For a probability measure $p\in P_{\mathrm{PCSFT}}$ and a variable $f\in V_{\mathrm{PCSFT}},$ we have

(13) $${\langle f\rangle }_{p}=\int_{H}f\left(\phi \right)dp\left(\phi \right)=\mathrm{Tr}{\hat{A}}_{f}{\hat{B}}_{p},$$

where $f\left(\phi \right)=\langle \phi |{\hat{A}}_{f}|\phi \rangle .$ This formula is obtained through expansion of the quadratic form $\langle \phi |{\hat{A}}_{f}|\phi \rangle$ w.r.t. the basis of eigenvectors of the Hermitian operator ${\hat{A}}_{f}.$

Let us consider the following maps $J_{S}:P_{\mathrm{PCSFT}}\to S_{\mathrm{QM}}$ and $J_{V}:V_{\mathrm{PCSFT}}\to V_{\mathrm{QM}}$,

(14) $$J_{S}\left(p\right)={\hat{\rho }}_{p}={\hat{B}}_{p}/\mathrm{Tr}B_{p},\;J_{V}\left(f\right)={\hat{A}}_{f}.$$

This correspondence connects the averages given by the causal theoretical and observational models:

(15) $$\frac{1}{E_{p}}{\langle f\rangle }_{p}=\mathrm{Tr}{\hat{\rho }}_{p}{\hat{A}}_{f};$$

i.e., the QM and PCSFT averages are coupled with the scaling factor, which is equal to the inverse of the average energy of the random field (for $H=L_{2}).$

Thus, density operators representing quantum states correspond to covariance operators of random fields normalized by the average energy of a random field, and the Hermitian operators representing quantum observables correspond to quadratic forms of fields.

Let us rewrite (15) in the form

$$\langle \frac{f}{E_{p}}\rangle_{p}=\mathrm{Tr}{\hat{\rho }}_{p}{\hat{A}}_{f}.$$

If random fields have low energy, i.e., $E_{p}<<1,$ the quantity

$$g_{p}\left(\phi \right)\equiv \frac{f(\phi )}{E_{p}}$$

can be interpreted as an amplification of the PCSFT physical variable f. Hence, by connecting QM with PCSFT, QM can be interpreted as an observational theory describing averages of amplified ‘subquantum’ physical quantities—quadratic forms of random fields. The subquantum random fields are unobservable and they can be experimentally verified only indirectly, via coupling with the observational model—QM.

In contrast to QM, PCSFT is causal: selection of a vector (‘field’) ϕ∈H determines the values of all PCSFT-quantities, quadratic forms of classical fields: $\phi \to \langle \phi |\hat{A}|\phi \rangle .$

For physical quantities, the correspondence map $J_{V}$ is one-to-one, but the map $J_{S}$ is not one-to-one. However, it is a surjecion; i.e., it is onto map.

## 5. Schrödinger Equation: QM vs. PCSFT

Now, we turn to the mathematical description of the subquantum field dynamics. First, we consider the Schrödinger equation in the standard QM formalism:

(16) $$ih\frac{\partial \psi }{\partial t}\left(t\right)=\hat{H}\psi \left(t\right),$$

(17) $$\psi \left(t_{0}\right)=\psi_{0},$$

where $\hat{H}$ is Hamiltonian.

In PCSFT, the same equation describes the dynamics of the subquantum field. Set $\hat{U}\left(t\right)=e^{-i\hat{H}/h};$ this is the unitary group determining the subquantum field dynamics; i.e., the initial field $\psi_{0}\in H$ evolves as $\psi \left(t\right)=\hat{U}\left(t\right)\psi_{0}.$ In causality’s terms, $\psi_{0}$ is the cause and ψ(t) is the effect. (Consideration of Hilbert space of square integrable functions, $H=L_{2}\left(R^{3};C\right),$ justifies the use of the term “field”).

We recall that a time-dependent random variable ϕ(t;ω) is called a *stochastic process*; we consider processes valued in complex Hilbert space H. In real physical modeling, $H=L_{2}.$ The dynamics of the prequantum random field (H-valued random variable) is described by the simplest stochastic process, which is provided by *deterministic dynamics with random initial conditions.*

In PCSFT, the Schrödinger equation, but with the random initial condition, describes dynamics of the subquantum random field; i.e., the subquantum stochastic process can be obtained from the mathematical equation that is used in QM for dynamics of the wave function:

(18) $$ih\frac{\partial \phi }{\partial t}\left(t;\omega \right)=\hat{H}\phi \left(t;\omega \right),$$

(19) $$\phi \left(t_{0};\omega \right)=\phi_{0}\left(\omega \right),$$

where the initial random field $\phi_{0}\left(x,\omega \right)$ is determined (but not uniquely) by the quantum pure state $\psi_{0}$ as follows. Consider the corresponding density operator ${\hat{\rho }}_{\psi_{0}}=|\psi_{0}\rangle \langle \psi_{0}|.$ Consider now a random field $\phi_{0}\left(\omega \right)$ with the covariance operator ${\hat{B}}_{\phi_{0}}$ such that $J_{S}\left({\hat{B}}_{\phi_{0}}\right)={\hat{B}}_{\phi_{0}}/\mathrm{Tr}{\hat{B}}_{\phi_{0}}={\hat{\rho }}_{\psi_{0}}.$ So, the quantum state dynamics t→ψ(t) is the formal mathematical image of a variety of subquantum dynamics corresponding to a variety of initial random fields that are encoded in QM by the same quantum state.

We remark that, up to the normalization factor, the covariance operator of the subquantum random field ϕ(t;ω) coincides with the density operator ${\hat{\rho }}_{\psi (t)}=|\psi \left(t\right)\rangle \langle \psi \left(t\right)|.$ Hence, the PCSFT and QM dynamics match each other, but only on the level of covariance vs. density operators.

The random field evolution is represented as

(20) $$\phi \left(t;\omega \right)=\hat{U}\left(t\right)\phi_{0}\left(\omega \right).$$

First, we remark that, if the mean value of the initial random field equals to zero, $E[\phi_{0}]=0,$ then E[ϕ(t)]=0, for any t. Finiteness of the second momentum is also preserved,

(21) $$E[||\hat{U}{\left(t\right)\phi ||}^{2}]=E[||\phi_{0}{\left|\right|}^{2}]<\infty .$$

Thus, $\phi :\left[0,+\infty \right)\to R_{\mathrm{PCSFT}}.$

By (21), we have

(22) $$\mathrm{Tr}{\hat{B}}_{\phi (t)}=E[||\hat{U}{\left(t\right)\phi ||}^{2}]=E[||\phi_{0}{\left|\right|}^{2}]=\mathrm{Tr}{\hat{B}}_{\phi_{0}};$$

i.e., the normalization factor in the map $J_{S}$ does not depend on t.

The previous considerations based on matching of the Schrödinger dynamics of pure quantum states and subquantum random fields can be generalized to matching of the von Neumann dynamics of a density operator and subquantum random field.

## 6. Interpretations of Quantum Mechanics and the Bild Conception

QM is characterized by the diversity of interpretations. Some experts consider this situation as a sign of the crisis in quantum foundations. Others struggle to convince the community that there can be found the “true interpretation” of QM. Pragmatic researchers typically proceed without even thinking about interpretations. Some experts claim that all interpretations have equal rights and it is merely a researcher’s test that determines the interpretation in regarding use. In any event, the problem of possible interpretations of QM is very complicated.

There are many shades of interpretation, but, following Ballentine [69,70,71], we wish to distinguish only two:

- **SI** *The statistical interpretation, according to which a pure state (and hence also a general state) provides a description of certain statistical properties of an ensemble of similarly prepared systems but need not provide a complete description of an individual system.*
- **II** *Interpretations that assert that a pure state provides a complete and exhaustive description of an individual physical system (e.g., an electron).*

We call **II** *the individual interpretation* of a quantum state. This interpretation is especially clearly presented in von Neumann’s book [42]. It is commonly known as the *orthodox Copenhagen interpretation.* The latter term has a variety of flavors, as was pointed out by Plotnitsky [30]; it is more natural to speak about interpretations in the spirit of Copenhagen. Unfortunately, Bohr expressed his views not so clearly. One might even guess that he used **SI** (see [34]). Moreover, the following terminological ambiguity contributed to the interrelation **II** vs. **SI** debate. The majority of **II** users would also tell that they use the statistical interpretation and accept that QM produces only statistical prediction. They combine (in some unclear for me) way the individual interpretation of a quantum state and the probabilistic description of experiments’ outcomes. For example, von Neumann suggested to describe “quantum probabilities” by using the von Mises frequency probability theory. However, how can the quantum state (as the state of say an individual electron) generate a random sequence (a collective in von Mises terminology)? In reply to this question, one of the paper’s reviewers wrote:

In contemporary quantum measurement theory, this role is attributed to the macroscopic apparatus which, coupled to the measured system, inevitably shares the random fluctuations of its state with the measured system, reflected in the outcome and the state after the measurement. That is, the statistical nature of the observed state is inherited from the macroscopic environment.

This viewpoint on the origin of quantum randomness justifies the use of the orthodox Copenhagen interpretation. However, it should be discussed in more detail, and, as was mentioned, we do not want to go deeper into the foundational discussions.

We remark that **SI** was actively advertized, e.g., by Blokhintsev [72,73], Ballentine [69,70,71], and De Muynck [74].

In fact, in Section 4, we employed **SI** without even mentioning the interpretation issue. Within **SI**, the statistical models similar to PCSFT are natural, as in combining the subquantum causality with the probabilistic structure of QM.

By employing **II** and especially following von Neumann [42], one might extract from QM a causal component. This is the model that solely handles quantum states and their Schrödinger dynamics. Mathematically, the quantum CTM is given by the space of quantum states $S_{\mathrm{QM}},$ the space of density operators. The corresponding OM is provided by the space of Hermitian operators $V_{\mathrm{QM}}.$ However, such split of QM into two counterparts and treatment of them as two separate models seems to be inconsistent even with von Neumann’s viewpoint, who considered QM as the integral model of quantum phenomena. Schrödinger would neither consider such a split of QM as an application of the Bild conception. Moreover, Einstein in his search for a true subquantum model was looking for something different from the aforementioned possibility to combine the Bild conception with **II**.

## 7. On Usefulness of Causal Theoretical Models

The above presentation of the possible two-level description of the microphenomena, QM vs. PCSFT, can be used as the initial point for the discussion on usefulness of CTMs. To be provocative, we start by noting that, for Bohr and other fellows of the orthodox Copenhagen interpretation of QM, attempts to construct CTM for QM are meaningless [23,29,30,31,32,33,34,35,75]. At the same time, Bohr never claimed that such a CTM cannot be constructed [31,33]; he was not interested in no-go theorems. In his writings, I did not find any word about the von Neumann no-go theorem. I am sure that he would ignore the Bell no-go theorem [28] and be surprised by the interest in it in the modern quantum foundational community. Bohr highlighted the observational status of QM, but, for him, any kind of CTM is metaphysical. For a “real physicist”, it is meaningless to spend time by trying to design a prequantum CTM. This position is very common among “real physicists”. For Bohr, it is impossible to complete QM in the causal way by operating with quantities that have direct connections to observations. Moreover, he is completely right: the complementarity principle and Heisenberg uncertainty relation block searching of a finer OM for QM. It seems that, in contrast to von Neumann, Bohr was not disturbed by acausality; observational acausality is a consequence of contextuality and complementarity of quantum observations. We also repeat that Bohr did not deny the possibility to construct CTMs beyond QM, but, for him, the introduction of hidden variables was a metaphysical and totally meaningless exercise.

Bohr’s position can be questioned, and on the questioners’ side are Helmholtz, Hertz, Boltzmann, and Schrödinger. As we have seen, Schrödinger agreed with Bohr that QM is a good OM for microphenomena. He did not think that the acausal structure of QM prevents construction of a corresponding CTM. For him, causality is closely coupled with continuity and hence to his original wave approach to microphenomena.

As Helmholtz, Hertz, Boltzmann, and Schrödinger, I think that consideration of acausality as a property of nature (at least at the micro level) completely destroys the methodology of science. If Helmholtz did make a mistake by saying [3] that every law of nature asserts that upon preconditions alike in a certain respect, there always follow consequences which are alike in a certain other respect, then physics becomes a science about gambling (as, e.g., QBists claim). It is difficult (at least for me) to accept this position. Thus, the main impact of creation of CTMs is reestablishing of causality that might be violated in OM.

Now, we turn to QM. Reestablishing of causality of microphenomena (even without the direct coupling to observations) would demystify quantum theory. We do not claim that PCSFT is the “true CTM” for QM; as Schrödinger claimed [10], it is meaningless and even dangerous for science development to search for such a model. However, PCSFT can be used as a causal Bild of quantum processes. One of the main advantages of this Bild is that it is local. PCSFT reproduces not only QM averages but event regarding its correlations [24]; hence, the Bell inequalities can be violated for PCSFT quantities (hidden variables from the observational viewpoint). PCSFT demystifies quantum entanglement by connecting it with correlations of subquantum (classical) random fields (cf. [24,25,26]). PCSFT can be considered as a step towards merging of QM with general relativity, but within some CTM.

Can one earn from CTM something that might be lifted to the observational level? In our concrete case, can some theoretical elements of PCSFT be realized experimentally (perhaps in the future)? The basic element of PCSFT is a random field ϕ=ϕ(x;ω). Measurement of such a subquantum field would be the real success of PCFT. However, it seems that one cannot expect this. As was pointed out by Bohr (in the 1930s), even the classical electromagnetic field cannot be measured in a fixed point.

Another component of PCSFT that can be connected with real physics is the need for the background field. This component was not discussed in the above brief presentation, so see [24,68] for details. Such a random background field $\phi_{\mathrm{bground}}\left(x,\omega \right)$ is the necessary element of the mathematical model $M_{\mathrm{PCSFT}}$ for generation of entangled states in $S_{\mathrm{QM}}.$ In this way, PCSFT is related to stochastic electrodynamics and supports it. Unfortunately, the background (zero point field) is not a component of conventional QM; stochastic electrodynamics is commonly considered an unconventional model of microphenomena. From the Bild viewpoint, this model should be treated as one possible CTM for QM; this viewpoint would clarify the interrelation between these two models. However, in this paper, we do not plan to go deeper into this issue. We note the background field carries long-distance correlations that contribute to violation of the Bell-type inequalities. However, these are classical field correlations having nothing to do with the spooky action at a distance.

The most close to experimental verification is PCSFT representation of Born’s rule as an approximate rule for calculation of probabilities; the standard Born rule is perturbed by additional terms that can in principle be verified (see [24] and especially article [68], suggesting a concrete experimental test). For a totally different reason, this prediction was tested by the research group of Prof. Weihs [76,77]—testing Sorkin’s inequality in the triple slit experiment. Surprisingly, transition from two slits to three slits is not trivial and Weihs’ group confronted difficulties related to nonlinearity of detection processes. For the moment, no deviations from the Born rule were observed.

For me, the main message of PCSFT as one possible CTM for QM is that “quantum nonlocality” is an artifact of OM (=QM). The presence of such artifacts in OMs is natural from the Bild viewpoint. This is one of the reasons to construct CYMs.

## 8. Bell’s Project from the Bild Viewpoint

Unfortunately, Bell did not read the works on the Bild conception for scientific theories. By introducing hidden variables, he suggested a special CTM for QM treated as OM. However, he considered coupling of his CTM $M_{\mathrm{Bell}}$ with OM $M_{\mathrm{QM}}$ as too special. The subquantum quantities, functions of hidden variables, A=A(λ), were identified with quantum observables. In particular, the ranges of values of quantities from $M_{\mathrm{Bell}}$ coincide with the ranges of values of quantum observables (and this is not the case, e.g., in PCSFT). As was pointed out in article [78], $M_{\mathrm{Bell}},$ confronts the complementarity principle. The latter point will be clarified below.

We recall the mathematical structure of $M_{\mathrm{Bell}}$ by connecting it with the framework of Section 3. Bell considered [28] an arbitrary set of hidden variables Λ; this is the set of states of his CTM; i.e., $S_{Bell}=\Lambda .$ To put this model in the mathematical framework of probability theory, Λ should be endowed with some σ-algebra of its subsets, say F. Denote by $P_{Bell}$ the space of all probability measures on (Λ,F). The space of subquantum quantities consists of all measurable functions A:Λ→R,A=A(λ), i.e., random variables in terms of the Kolmogorov probability theory. We stress that the correspondence map $J_{S}:P_{Bell}\to S_{QM}$ is not specified; it is just assumed that such a map exists. For Bell’s reasoning [28], this map need not be onto $S_{QM}$ (so it need not be a surjection).

To make his model with hidden variables straightforwardly experimentally verifiable, Bell identified the values of CTM quantities A=A(λ) with the values of QM quantities, the outcomes of quantum observables. First, we discuss the mathematical side of this assumption and then its foundational side.

Mathematically, identification of quantities of $M_{\mathrm{Bell}}$ with QM observables means that the range of values of $A\in V_{Bell}$ coincides with the spectrum of the corresponding Hermitian operator $\hat{A}.$ This is the important mathematical constraint on the map $J_{V}:V_{Bell}\to V_{QM}$ (we recall that $V_{QM}$ is the set of density operators). Purely mathematical relaxation of this assumption destroys the Bell inequality argument, e.g., as in PCSFT.

However, Bell *should* proceed with this assumption on the coincidence of ranges of values of the subquantum quantities and quantum observables since he dreamed of straightforward experimental verification of his model with hidden variables [27,28]. He was not accustomed to the Bild concept of a scientific theory. In particular, Hertz’s (and Schrödinger’s) statement on hidden quantities that could not be observed directly was totally foreign for Bell. For him, as well as for Bohr, a theory in which quantities cannot be directly verified is a part of metaphysics, not physics [23,29,30,31,32,33,34,35,75].

By identifying the outcomes of subquantum quantities with the outcomes of quantum observables, Bell confronts the complementarity principle. This can be clearly seen in the CHSH-framework [79]. There are considered two pairs of observables: $(A_{1},A_{2}),$ in “Alice’s lab”, and $(B_{1},B_{2}),$ in “Bob’s lab”, represented by Hermitian operators $\left({\hat{A}}_{1},{\hat{A}}_{2}\right)$ and $({\hat{B}}_{1},{\hat{B}}_{2}).$ Observables corresponding to cross measurements for Alice–Bob are compatible; i.e., they can be jointly measurable, but local observables of Alice as well as of Bob are incompatible; i.e., they cannot be jointly measurable. In the operator terms,

(23) $$\left[{\hat{A}}_{i},{\hat{B}}_{j}\right]=0,\;\left[{\hat{A}}_{1},{\hat{A}}_{2}\right]\neq 0,\;\left[{\hat{B}}_{1},{\hat{B}}_{2}\right]\neq 0.$$

This is the quantum mechanical description of the CHSH experimental context. We note that, if local observables are compatible for at least one lab (in the operator terms, at least one of commutators $\left[{\hat{A}}_{1},{\hat{A}}_{2}\right],\left[{\hat{B}}_{1},{\hat{B}}_{2}\right]$ equals to zero), then the CHSH inequality cannot be violated [78]. We explain this point in more detail.

For quantum observables, the correlation used in the CHSH framework can be represented via the Bell operator:

(24) $$\hat{B}=\frac{1}{2}\left[{\hat{A}}_{1}\left({\hat{B}}_{1}+{\hat{B}}_{2}\right)+{\hat{A}}_{2}\left({\hat{B}}_{1}-{\hat{B}}_{2}\right)\right]$$

as

(25) $${\langle B\rangle }_{\psi }=\langle \psi |\hat{B}|\psi \rangle .$$

By the Landau identity, we have

(26) $${\hat{B}}^{2}=I-\left(1/4\right)\left[{\hat{A}}_{1},{\hat{A}}_{2}\right]\left[{\hat{B}}_{1},{\hat{B}}_{2}\right].$$

Hence, if *at least one of the commutators* is zero,

(27) $$[{\hat{A}}_{1},{\hat{A}}_{2}]=0,$$

or

(28) $$[{\hat{B}}_{1},{\hat{B}}_{2}]=0,$$

then the following inequality holds:

(29) $${\left|\langle B\rangle \right|}_{\psi }\leq 1.$$

We remark that such purely quantum mechanical treatment of the Bell-type inequalities does not involve the issue of nonlocality at all. The crucial point of the above consideration is the existence of incompatible observables. The latter is a consequence of the Heisenberg uncertainty principle or more generally the Bohr complementarity principle (see [78,80,81] for the extended discussions).

Bell considered quantities of his CTM $M_{\mathrm{Bell}}$ as representing physical observables; hence, all observables can be represented as functions $A_{i}=A_{i}\left(\lambda \right),B_{j}=A_{i}\left(\lambda \right)$ and their values are identified with outcomes of observations. Aside from the pairs $\left(A_{i}\left(\lambda \right),B_{j}\left(\lambda \right)\right)$ of compatible observables, one can consider the pairs $\left(A_{i}\left(\lambda \right),A_{j}\left(\lambda \right)\right)$ and $(B_{i}\left(\lambda \right),B_{j}\left(\lambda \right)).$ By treating the latter two pairs as representing the outcomes of physical observables, it is natural to assume that their joint measurability may not be possible now but in the future, when the measurement technologies would be improved. So, the complementarity principle loses its fundamental value. By keeping Bell’s model $M_{\mathrm{Bell}}$ as representing physical reality, one confronts treating of complementarity as the fundamental property of (observational) microphenomena.

At the level of correlations,

(30) $${\langle A_{i}B_{j}\rangle }_{\rho }=\mathrm{Tr}\hat{\rho }{\hat{A}}_{i}{\hat{B}}_{j}=\lim_{N\to \infty }\frac{1}{N}\sum_{k=1}^{N}A_{ik}B_{jk},$$

where $\left(A_{ik}\right),\left(B_{jk}\right)$ are observables’ outcomes. At the same time, for the probability distribution $P_{\rho }$ such that $\hat{\rho }=J_{S}\left(P_{\rho }\right),$ we have

(31) $${\langle A_{i}B_{j}\rangle }_{P_{\rho }}=\int_{\Lambda }A_{i}\left(\lambda \right)B_{j}\left(\lambda \right)P_{\rho }\left(d\lambda \right)=\lim_{N\to \infty }\frac{1}{N}\sum_{k=1}^{N}A_{ik}^{\prime }B_{jk}^{\prime }$$

where $A_{ik}^{\prime }$ and $B_{jk}^{\prime }$ are outcomes of random variables $A_{i}=A_{i}\left(\lambda \right)$ and $B_{j}=B_{j}\left(\lambda \right).$ However, since these outcomes can be identified with the outcomes of the quantum observables, $A_{ik}^{\prime }=A_{ik},B_{jk}^{\prime }=B_{jk},$ we can write

(32) $${\langle A_{i}B_{j}\rangle }_{P_{\rho }}=\lim_{N\to \infty }\frac{1}{N}\sum_{k=1}^{N}A_{ik}B_{jk}.$$

However, the same reasoning is applicable to the subquantum random variables $A_{1}=A_{1}\left(\lambda \right)$, $A_{2}=A_{2}\left(\lambda \right)$, and $B_{1}=B_{1}\left(\lambda \right),B_{2}=B_{2}\left(\lambda \right)$, representing the incompatible quantum observables:

(33) $${\langle A_{1}A_{2}\rangle }_{P_{\rho }}=\int_{\Lambda }A_{1}\left(\lambda \right)A_{2}\left(\lambda \right)P_{\rho }\left(d\lambda \right)=\lim_{N\to \infty }\frac{1}{N}\sum_{k=1}^{N}A_{1k}^{\prime }A_{2k}^{\prime },$$

(34) $${\langle B_{1}B_{2}\rangle }_{P_{\rho }}=\int_{\Lambda }B_{1}\left(\lambda \right)B_{2}\left(\lambda \right)P_{\rho }\left(d\lambda \right)=\lim_{N\to \infty }\frac{1}{N}\sum_{k=1}^{N}B_{1k}^{\prime }B_{2k}^{\prime }.$$

Again, by identifying the values of subquantum and quantum observables, we obtain

(35) $${\langle A_{1}A_{2}\rangle }_{P_{\rho }}=\lim_{N\to \infty }\frac{1}{N}\sum_{k=1}^{N}A_{1k}A_{2k},$$

(36) $${\langle B_{1}B_{2}\rangle }_{P_{\rho }}=\lim_{N\to \infty }\frac{1}{N}\sum_{k=1}^{N}B_{1k}B_{2k}.$$

Representations (35) and (36) of subquantum correlations (within CTM $M_{\mathrm{Bell}})$ via outcomes of observables support the assertion that the subquantum correlations ${\langle A_{1}A_{2}\rangle }_{P_{\rho }},{\langle B_{1}B_{2}\rangle }_{P_{\rho }}$ should be measurable (at least in principle and in the future). It is not clear how Bell would treat this objection to his argument. I guess that he would agree that his model with hidden variables, $M_{\mathrm{Bell}},$ collides with the complementarity principle. However, he might choose to move between Scylla and Charybdis:

- **S**: identification of the values of subquantum random variables with the values of quantum observables;
- **Ch**: the complementarity principle;

and claim that **S** and **Ch** can peacefully coexist. He might say that (32) is legal and hence the experimental verification of $M_{\mathrm{Bell}}$ is possible, but (35) and (36) are illegal and treatment of these correlations as experimentally verifiable is forbidden. For me, the latter position is inconsistent (although logically possible). This inconsistency was the basis of De Broglie’s critique of Bell’s argument [36] (Section 9).

This is a good place to recall that the physical seed of the complementarity principle is in Bohr’s quantum postulate on the existence of indivisible quantum of action given by the Planck constant h: incompatible observables exist due to the existence in nature of minimal action [82,83,84] (see [81]). Thus, Bell’s conflict with the complementarity principle is in fact the conflict with the quantum postulate—the existence of h. Hence, this is a conflict with the very foundation of quantum physics, e.g., the quantum model of black body radiation and processes of spontaneous and stimulated emissions.

We conclude this section by noticing that PCSFT reproduces quantum correlations (see [24]), and, hence, the PCSFT correlations violate the Bell inequalities. However, this is not surprising since the ranges of the values of the subquantum quantities (represented as quadratic forms of the fields) and quantum observables (represented as Hermitian operators) do not coincide. Moreover, this is a good place to recall that the proofs of the Bell-type theorems are based on the assumption that the functions of hidden variables are valued in in the segment [−1,+1].

## 9. De Broglie’s Critique of No-Go Theorems of Von Neumann and Bell

Nowadays, it is practically forgotten that De Broglie considered all no-go theorems by which hidden variables for QM do not exist as totally misleading [36]. His double solution model [40] can be considered as a model with hidden variables of the field type. The pilot wave is a hidden variable. In this aspect, the double solution model and PCSFT are similar. Schrödinger’s attempt to find a subquantum model of the wave type as well as Einstein’s search for a subquantum nonlinear field model were also steps in the same direction, although, as was noted, methodologically, their positions are different. Schrödinger tried to realize the Bild approach for QM, treated as OM, but Einstein dreamed of a model jointly carrying the features of CTM and OM. As we shall see, De Broglie followed the Bild conception without knowing about it.

To justify his double solution model and its peaceful coexistence with QM, De Broglie criticized the most famous no-go theorems, the von Neumann and Bell theorems [28,42]. He did not criticize the mathematical derivation of these theorems but their interpretation and straightforward identification of hidden and observational quantities. His interpretation of these theorems was presented in great detail by Lochak [37,38,39]. Paper [38] is available for free reading via Google Books; we cite it as follows:

Von Neumann proved a theorem which claims that there are no pure states without statistical dispersion. This result is indeed intuitively obvious because the absence of dispersion in a pure state mean that it would be possible to measure simultaneously the physical quantities attached to a system described by this state. But in fact we know that this is impossible for non-commuting quantities. In this sense, the theorem is nothing but a consequence of Heisenberg’s uncertainties.

From this, one can conclude that a pure state of QM cannot be considered as representing an ensemble of systems following the laws of classical probability theory (see [38] for detailed discussion) and quantum observables as classical random variables. From this, von Neumann reached the conclusion that, generally, it is impossible to create any model with hidden variables behind QM, as pointed out in [38].

De Broglie’s answer consists essentially in asserting that if any hidden parameters do exist, they cannot obey quantum mechanics because if you try to imagine hidden parameters it is of course in order to restore the classical scheme of probabilities. Now if you need a classical scheme of probabilities for objective (but hidden) values of physical quantities which are introduced in quantity of hidden parameters, these probabilities cannot be probabilities observed on the result of a measurement: simply because the observed probabilities do obey the quantum scheme and not the classical one!

Hence, not only hidden parameters λ∈Λ but even quantities A=A(λ) are hidden, and the probability distributions of these quantities $P_{A}$ should not be identified with quantum probability distributions.

For De Broglie, it was evident that classical and quantum probability calculi differ crucially; by attempting to apply the former for quantum observables, one is immediately confronted with the Heisenberg uncertainty principle (and Bohr’s complementarity principle [23,29,30,31,32,33,34,35]). This is precisely my viewpoint, which was presented in article [80] (see Section 8).

Hence, De Broglie’s viewpoint on the interrelation of subquantum and quantum models matches the CTM-OM approach perfectly. In fact, it matches the ontic–epistemic framework since De Broglie considered hidden variables and physical quantities, A=A(λ), as objective entities. However, as was already noted, schematically, CTM-OM and the ontic–epistemic frameworks are similar.

De Broglie’s statement that quantities of a subquantum theory are hidden and their probability distributions should not be identified with the probability distributions of quantum observables matches the PCSFT-QM coupling considered in Section 4. PCFT quantities are quadratic forms of fields playing the role of hidden variables. Such quantities have a continuous range of values, but say the quantum spin observables have the discrete spectra. Of course, they cannot have the same probability distribution, even without consideration of correlations. The correspondence between classical probability calculus of PCSFT and the quantum probability calculus is fuzzy. Classical covariance operators are mapped to density operators; see (14).

De Broglie and Lochak used the same argument for the critical analysis of the Bell theorem: one should sharply distinguish between subquantum and quantum quantities and not identify their outcomes and probability distributions. Not only are hidden parameters hidden but also quantities dependent on them and their probability distributions. So, Bell’s model is a very special CTM for QM. Yes, it should be rejected, as follows from the quantum formalism and experiments. However, its rejection does not prevent search for other CTMs for QM with more complicated connections between subquantum and quantum quantities and their probability distributions. From this viewpoint, the foundational value of the Bell theorem is overestimated.

I again repeat that it is a pity that the fathers of QM, including De Broglie, were not aware of the works of Helmholtz, Hertz, and Boltzmann. The Bild conception would provide the rigid philosophic basis for establishing proper interrelation between subquantum models with hidden variables and QM.

## 10. Classical Mechanics

For classical mechanics, CTM and OM coincide. On one hand, this was fortunate for the development of physics since it simplified its philosophic basis so much and highlighted the role of observation. On the other hand, identification of one special mathematical model, Newtonian mechanics, with reality supported similar ontic treatment of all physical models. The ontic viewpoint of a scientific theory dominated for a few hundreds years, up to the works of Helmholtz, Hertz, Boltzmann, and Schrödinger. However, these works did not revolutionize the philosophy of science. For example, acausality of QM is still considered as a property of nature such that irreducible quantum randomness is ontic.

We note that it seems that Hertz did not consider classical mechanics as OM [2]: If we try to understand the motions of bodies around us, and to refer them to simple and clear rules, paying attention only to what can be directly observed, our attempt will in general fail.

This statement definitely refers to classical mechanics. Similar to Hertz, Atmanspacher [56] also considered the two-level description even for classical physics and suggested the corresponding mathematical examples.

The need for a separate OM model for classical mechanics became clear in the process of creation of the mathematical description of the Brownian motion, which will be considered in the next section, and CTM-OM structured in accordance with the Bild conception.

### 10.1. Brownian Motion: Two Levels of Description from the Timescale Separation

Here, we follow the article of Allahverdyan, Khrennikov, and Nieuwenhuizen [45]:

The dynamics of a Brownian particle can be observed at two levels [85]. Within the first, more fundamental level the Brownian particle coupled to a thermal bath at temperature *T* is described via definite coordinate *x* and momentum *p* and moves under influence of external potential, friction force, and an external random force. The latter two forces are generated by the bath. The second, overdamped regime applies when the characteristic relaxation time of the coordinate $\tau_{x}$ is much larger than that of the momentum $\tau_{p}$,

$$\tau_{x}\gg \tau_{p}$$

(overdamped regime). On times much larger than $\tau_{p}$ one is interested in the change of the coordinate and defines the *coarse-grained* velocity as v=Δx/Δt for $\tau_{x}\gg \Delta t\gg \tau_{p}$. This definition of *v* is the only operationally meaningful one for the (effective) velocity within the overdamped regime. It appears that the coarse-grained velocity, though pertaining to single particles, is defined in the context of the whole systems of coupled Brownian particles.

The evolution of the momenta of the Brownian particles is very fast and cannot be resolved on the timescales available to the experiment. To obtain experimentally accessible quantities, one employs the technique of the timescale separation and measurement of the coarse-grained velocity and osmotic velocity. These quantities can be measured. They are assigned not to an individual Brownian particle but to an ensemble of particles coupled to both, so these are statistical quantities.

In terms of the present article, Brownian motion is described by CTM $M_{CB}$ with the phase space (x,p) and OM $M_{\mathrm{OB}}$ with the coarse-grained velocities $v_{+},v_{-}$ or the osmotic velocity $u=v_{+}-v_{-},$ The later description is based on observational quantities (x,u). As was shown in article [45], $M_{\mathrm{OB}}$ shows some properties of QM; e.g., there are analogs of the Heisenberg uncertainty relations and entanglement; in particular, for a pair of Brownian particles, the joint probability distribution $P(t,x_{1},u_{1},x_{2},u_{2})$ does not exist. Of course, the OMs $M_{\mathrm{OB}}$ and $M_{\mathrm{QM}}$ differ essentially. For example, for a single particle, the probability distribution P(t,x,u) is well-defined; incompatibility appears only in compound systems.

Nowadays, the above two-level structuring of the scientific theory of Brownian motion is shaken by the novel experimental possibilities for the measurement of momentum *p* of a Brownian particle. A variety of experiments were performed during the last few years (see, e.g., [86]). In spite of some diversity in experimental outputs, it is clear that experimental science is on the way to establishing the robust procedures for measurement of the Brownian momentum *p*. Through experimental research, CTM $M_{\mathrm{CB}}$ is achieving OM status. However, new theoretical efforts are needed to merge $M_{\mathrm{CB}}$ and $M_{\mathrm{OB}}$ treated as OMs. The osmotic velocity *u* (an element of $M_{\mathrm{OB}})$ is not straightforwardly derived within $M_{\mathrm{CB}}.$ At least for me, the connection between the velocity and coarse-grained velocity is not clear. How is the latter derived from the former?

This special example supports the search for CTMs for QM (see the discussion at the end of Section 7). Some hidden quantities of such models can serve as the candidates for the future experimental verification. One of the problems of such a project is that, since the creation of QM, physicists (mathematicians and philosophers) created too many subquantum models operating with a variety of hidden quantities as say the quantum potential in Bohmian mechanics or the random field in PCSFT. What are the most probable candidates for future experimental verification? The Bell hidden variable model [27,28] is one of the CTMs for QM; it can be directly tested experimentally. It was tested and rejected.

## 11. Discussion on the Bild Conception and Its Role in Foundations of Science

My aim is to recall to physicists and especially to experts in foundations (not only of quantum physics but also classical mechanics and field theory, statistical mechanics, and thermodynamics) about the works of Helmholtz, Hertz, and Boltzmann [1,2,3,7,8] on the meaning of a scientific theory that led to the Bild conception—the mathematical model concept of a scientific theory. By appealing to the two-level description of natural phenomena, CTM-OM description, it is possible to resolve many foundational problems, including acausality of QM. Moreover, the Bild conception demystifies quantum foundations. The “genuine quantum foundational problems” such as the possibility to introduce hidden variables were discussed long ago. The latter problem was analyzed by Hertz, who tried to reduce the classical electromagnetic field to an ensemble of mechanical oscillators [1]. From the viewpoint of the Bild conception, Bell’s attempt to invent hidden variables for QM is very naive; if such variables existed, their coupling with quantum observables might not be as straightforward as in the Bell model. Within the Bild conception, it becomes clear why Schrödinger did not consider acausality of quantum observations as the barrier on the way towards a causal description of quantum phenomena [9,10]. It seems that, similar to Bell [28], von Neumann was neither aware of the development of the philosophy of science by the German school of physicists in the 19th century. He treated the quantum measurement problem too straightforwardly, and acausality and irreducible quantum randomness appeared as consequences of such treatment [42]. He did not appeal to the two-level CTM-OM description of microphenomena.

In a series of works [24,25,66,67,68], Khrennikov et al. developed PCSFT, CTM with classical random fields, reproducing QM interpreted as OM for microphenomena. However, PSCFT-QM coupling is not so simple as in the Bell framework.

The two-level description of physical phenomena is in fact widely used in statistical physics and is based on a timescale separation technique and consideration of coarse quantities. All such descriptions are well accommodated within the Bild conception. The Brownian motion in the overdamped regime is described by OM, which is not directly coupled to CTM based on the classical mechanical description.

We point out that paper [22] also reviews Wittgenstein’s works, which are closely related to Hertz’s works. However, we would not consider Wittgenstein’s contribution to the development of the Bild conception [87,88].

Finally, we remark that the Primas–Atmanspacher [54,55] ontic–epistemic approach to physical theories (see also, e.g., [56,57]) is formally similar to the Bild conception. However, in accordance with the Bild concept, no model describes reality as it is.

## Data Availability

Data are contained within the article.

## Appendix A

Here, we follow the article of Allahverdyan, Khrennikov, and Nieuwenhuizen [45].

The system under analysis consists of *N* identical Brownian particles with coordinates $x=(x_{1},\ldots ,x_{N})$ and mass m; particles interact with thermal baths at temperatures $T_{i}$ and coupled via a potential $U(x_{1},\ldots ,x_{N})$. We consider so-called *overdamped limit* [85]:

- The characteristic relaxation time of particles’ momenta $p_{i}=m{\dot{x}}_{i}$ is essentially less than the characteristic relaxation time of the coordinates: $\tau_{x}\gg \tau_{p}.$
- Dynamics is considered in the time range:
  $$\tau_{p}<<t\leq \tau_{x}.$$

The conditional probability $P(x,t|x^{\prime },t^{\prime })$ satisfies the Fokker–Planck equation (the special case of the Kolmogorov equation for diffusion) [85]:

(A1) $$\partial_{t}P\left(x,t|x^{\prime },t^{\prime }\right)=-\sum_{i}\partial_{x_{i}}\;\left[\;f_{i}\left(x\right)\;P\left(x,t|x^{\prime },t^{\prime }\right)\;\right]+\sum_{i}T_{i}\;\partial_{x_{i}x_{i}}^{2}P\left(x,t|x^{\prime },t^{\prime }\right),\;t\geq t^{\prime },$$

with the initial condition (corresponding to the definition of conditional probability)

(A2) $$P\left(x,t|x^{\prime },t\right)=\delta \left(x-x^{\prime }\right)\equiv \prod_{i=1}^{N}\delta \left(x_{i}-x_{i}^{\prime }\right).$$

Now, consider an ensemble Σ(x,t) of all realizations of the *N*-particle system having at time *t* the fixed coordinate vector x. This ensemble of systems is chosen out of all possible realizations for measuring particles’ coordinates. For Σ(x,t), the average coarse-grained velocity for the particle with index *j* might be heuristically defined as

(A3) $$v_{j}\left(x,t\right)=\lim_{\epsilon \to 0}\;\int dy\;\frac{y_{j}-x_{j}}{\epsilon }\;P\left(y,t+\epsilon |x,t\right).$$

However, irregularity of the Brownian trajectories implies nonexistence of this limit, so one should define the velocities for different directions of time [89]:

(A4) $$v_{+,j}\left(x,t\right)=\lim_{\epsilon \to +0}\;\int dy_{j}\;\frac{y_{j}-x_{j}}{\epsilon }\;P\left(y_{j},t+\epsilon |x,t\right),$$

(A5) $$v_{-,j}\left(x,t\right)=\lim_{\epsilon \to +0}\;\int dy_{j}\;\frac{x_{j}-y_{j}}{\epsilon }\;P\left(y_{j},t-\epsilon |x,t\right).$$

What is the physical meaning of these expressions? The directional coarse-grained velocity $v_{+,j}\left(x,t\right)$ is the average velocity to move anywhere starting from (x,t), whereas $v_{-,j}\left(x,t\right)$ is the average velocity to come from anywhere and to arrive at x at the moment *t*.

For the overdamped Brownian motion, almost all trajectories are not smooth, and this is the reason for

(A6) $$v_{+,j}\left(x,t\right)\neq v_{-,j}\left(x,t\right).$$

The difference

(A7) $$u\left(x,t\right)=v_{+,j}\left(x,t\right)-v_{-,j}\left(x,t\right)$$

characterizes the degree of nonsmoothness; it is called osmotic velocity; analytically, it can be represented in the form

(A8) $$u_{j}\left(x,t\right)=\frac{v_{-,j}\left(x,t\right)-v_{+,j}\left(x,t\right)}{2}=-T_{j}\partial_{x_{j}}\ln P\left(x,t\right).$$

If we consider ϵ much smaller than the characteristic relaxation time of the momentum (apply definitions (A4) and (A5) to a smoother trajectory), then $v_{+,j}\left(x,t\right)$ and $v_{-,j}\left(x,t\right)$ will be equal to each other and equal to the average momentum.
